# Operative Dentistry

**Published:** 1895-08

**Authors:** 


### MONTHLY DIGEST.

### Operative Dentistry.

(*Continued from Page* 252.)

### OUR RESPONSIBILITIES.

DR. M. L. HANAFORD, in a paper read before the Northern Illinois Dental Society,* discusses the subject of the responsibilites which rest upon the dentist, toward his patients, toward the public, toward the profession and toward himself, which, if fully met requires "an array of virtue and strength of character most wonderfully comprehensive, toward which as our ideal though we may strive ever so faithfully, there will still be other and greater possibilities before us."

* Dental Review, Feb. 15, 1895.

HOW CAN WE CONVINCE OUR PATIENTS THAT WE ARE HUMAN? is the title of a paper read by Dr. M. R. Harned, before the Northern Illinois Dental Society.* According to the dictionary "*Human* is what every man is; *Humane* is what every man should be," and humane is clearly what Dr. Harned thinks dentists ought to be toward their patients, as evidenced by the suggestions that he offers for the palliation of the evils incident to the dental rack of torture." He says: "Our first and ever present idea should be to help humanity out of her affliction as far as possible. At least palliate the dread effects of inheritance and ignorance. If we teach the people not to dread us, then we teach them to take care of themselves as far as we know, and it should be our constant study to know more, and in helping them, we help ourselves. . . Our duties to humanity are not only to preserve teeth, but to conserve vital energy, and by scientific attainment deserve the title of *professional* men.

IN THE RUT, OR "WHERE AM I AT?"

Under this title, Dr. C. J. UNDERWOOD, in the Northern Illinois Dental Society† discusses four classes, in one or the other of which, dentists, as well as men in other ranks of life, may be found.

First, the ultra-conservative, or *fossil*, burrowing in what *was* the rut before it was buried under the *debris* from the wheels of progress. He knows nothing of disinfectants; he never fills root-canals, preferring a recut hole under the margin of the gum; "gum-sets" are his delight, for the insertion of which he tears out the abutments of the most promising bridge, and uproots the prospects of the most desirable crown. He is mostly bald and very gray and there are very few of him.

Second—The *conservative*—jogging on in the rut, holding on to that which is good, sailing by chart over known seas, and in safe harbors.

Third—The *radical*—breaking his neck to find a shorter cut or a smother track;

* Dental Review, Feb. 15, 1895.
† Dental Review, Feb. 15, 1895.

And, Fourth—The ultra-radical, the *crank*—egotistical, skeptical and unstable, imagining himself in the lead, swayed by the same spirit of selfish lust for gain and flattering notoriety that, in politics, makes the anarchist, in religion, the atheist.

As it takes all kinds of people to make a world, so it may be fair to assume that all of these characters are necessary to the best and most rapid development of our profession.

Let us hitch on to the professional chariot, and so unite, and blend effort and intelligence, that our progress may be marked by a rut that no one need be ashamed to travel in.

"AMBITION."

AMBITION is the subject of the leading editorial of the *Review.** The distinction is drawn between honorable, laudable ambition, or that which seeks aggrandizement at the expense of others.

The ambition for a young man to cultivate is that which urges him to accomplish something for the profession that no other man has ever been able to accomplish. The ambition which impels him to bring honor to his profession, and happiness to humanity, irrespective of personal aggrandizement, is the ambition which, after all, proves to be the surest road to a worthy prominence.

DR. CROUSE continued his "Suggestions on Developing and Conducting a Dental Practice on Business Principles."†

The points dwelt upon in the present chapter are cleanliness of person and surroundings, personal hygiene, self-control, and the means of minimizing pain—first in the application and retention of the rubber dam, the method adopted by the author for retention being the use of spunk and sandarac varnish, or a little oxyphosphate of zinc, applied to the face of the tooth; the use of clamps he considers neither necessary, wise nor humane.

To obtund sensitive dentine he advises the alternately repeated application of carbolic acid and tincture of aconite root, and the use of keen, sharp, modern burs—never using on sensi-

*Dental Review, February 15, 1895.

† Dental Digest, February, 1895.

tive dentine the burs which has been dulled by cutting enamel. He says in conclusion of this branch of his subject: "The dentist who inflicts unnecessary suffering or who neglects to relieve his patient from all suffering possible, by adopting every legitimate means, is certainly censurable and does not deserve any degree of success."

MODERATION IN PRACTICE AND IN STATEMENT.

Under this title DR. S. G. PERRY, in a paper read before the New York Odontological Society,* makes an earnest though somewhat humorous plea for more conservative accuracy in observation and expression, and a cultivation of the scientific spirit.

As long as we are careless in our habits of thinking, careless in the observation of facts and hasty in our deductions, careless in our methods and in our statements of experience and opinions, we cannot in justice claim that we are *a very scientific body of men*, or that our profession is a very exact one.

The most practical way of helping our profession to a higher plane lies in the encouragement of the scientific spirit, in exactness in practice and in the tabulation and report of our experience.

To eliminate, as far as possible, *the personal equation* is an acquirement of the first importance; the discovery of facts uninfluenced by fancies, using the careful, accurate language of science in describing them; saying exactly what is meant, and meaning exactly what is said; not confounding *opinions* with facts, though the opinions of those who have had long experience also have great value.

The same spirit of moderation should also be cultivated in modes of practice. The moderate man will be cautious about the promiscuous insertion of immovable bridges; in placing all-gold crowns in conspicuous places; in putting large gold contour in weak, shaky teeth. He will realize that the highest art is the art that conceals art. Finally, the dentist should be an all-around man; he should have many sides, and "moderation" ought to be stamped on every one of them.

*International Dental Journal, March, 1895.

### THE LOYALTY OF SOCIETY MEMBERS.

This topic is discussed editorially in the *International Dental Journal*,* the line of argument being that each member, and particularly those best endowed by natural capacity and acquired ability, should give to their local society their entire work in any given direction, otherwise the society is being deprived of strength and sources of growth, while the society receiving such assistance will fail to do efficient work when deprived of this stimulus.

Local, State and National societies should form an ascending scale; the work of individual members of local societies should be winnowed and sifted by the State Associations, and put into digestible form for presentation before the National bodies, where all advanced work should have final consideration.

The proceedings of the National Associations "qualify the world's opinion of the status of dental science and practice, and should present a criterion for the guidance of all."

The negative side of the question of "Raising the Entrance Standard" to dental colleges is presented editorially in the *International Dental Journal*.† In the words of Profs. Pierce and Taft, what is wanted is "a reasonable degree of scholarship," with more attention to the examinations throughout the course, especially the final one. Unless there is a known *moral* reason against it, admit them, and by giving each one thorough, conscientious training, instruction and care, endeavor to develop such men (and women?) as will fill their places in the field of dentistry with credit. "Raise the standard" by seeing that it embraces ethics and morals, for in the neglect of this lies the secret of much of the discredit that has come upon the profession

The editor of the *Dental Digest*‡ discusses briefly the subject of "Dental Legislation," taking the ground that when a diploma is required, the methods of the school ought to be open to inspection and examination at any time, and when a diploma is

*International Dental Journal, March, 1895.
†International Dental Journal, March, 1895.
‡Dental Digest, February, 1895.

not required, that a limit of at least three years' practical work ought to be exacted before application for license is entertained.

### DENTAL PROTECTIVE ASSOCIATION.*

The suit defended by the Association on the "Low Bridge Patent" has been indefinitely postponed owing to a press of other cases, and the ending of the term of the Federal Court in Brooklyn. Dr. Crouse thinks the case will come up for a final hearing in the near future.

The editor of the *Dental Digest*† calls attention to a recent curious decision of the Illinois State Board of Health, *viz.:* that a graduate of pharmacy, of veterinary science, or of dentistry, is not entitled to one year's standing in a medical college, but must enter as a freshman! As graduates of reputable dental colleges are entitled to membership in the American Medical Association, if members of a local medical society, there is an incongruity somewhere. The editor of the official journal of the American Medical Association, in discussing the matter, thinks that such discrimination "ought to lead to organizations . . . similar to the American College Association," quite ignoring the ten-year-old National Association of Dental Faculties.

### FRACTURES OF THE MAXILLA.

Dr. L. P. Haight‡ gives two methods of treatment of fractures of the inferior maxilla. The first method is very similar to the second method given by Dr. Gilmer, with the exception that articulation of the opposing bones is secured before taking the impression of the teeth for the splint. By the second method given by Dr. Haight, bands are cemented to a tooth on either side of the fracture (in the case given, the fracture extending up between the central incisors, the cuspids were banded). Tubes of German silver are soldered to the bands, through which a wire is passed, which is bent at a right angle at one end, and having a screw thread cut on the other end projecting beyond the band to receive a nut. The fractured ends are drawn together

*Dental Digest, February, 1895.
†Dental Digest, February, 1895.
‡ Items of Interest, February, 1895.

by tightening the nut on the screw end of the wire. Dr. Haight cautions against tight bandaging, avoiding the bandage entirely if possible, as the free and untrammeled supply of blood to the injured part is one of the secrets of success in securing perfect union of the fractured bone.

### THE ADAPTABILITY OF FILLING MATERIALS.

DR. C. E. FRANCIS, in an article read before the Midwinter Fair Dental Congress,* under the general heading, "Practical Suggestions," concludes that, while *solidity* is an essential feature in all fillings, *adaptability* of the material chosen to the cavity walls is absolutely necessary in all cases, and lack of this feature the most general cause of failure. This he attributes to the too general use of cohesive gold in places where soft, pliable stoppings, are demanded, and where the choice lies between soft gold and tin-foil. Pure tin, as prepared by Prof. Darby, in thin shavings of pure block tin, or the cohesive tin-foil, more recently manufactured by Kearsing, is recommended by Dr. Francis as a most excellent agent for securing safety to frail cavity walls, and for preserving them without further deterioration or loss of tooth structure.

DR. LORD, in the New York Odontological Society,† spoke of the tin foil recently brought out by Mr. Williams as superior to any he had ever used. It works waxy, so that almost anything can be done with it with suitable instruments. You can build out or contour with it, as much as you desire, if the cavity has three walls remaining. It is a No. 3 foil.

### COMBINATION FILLINGS.

DR. S. B. PALMER‡ explains the value of combination fillings from the standpoint of chemical and electrical science, showing the results of the combination of tin and gold in varying proportions, the relations of tin and amalgam, of amalgam and amalgam when joined at different times, and the combination of gold and amalgam.

* Dental Headlight, Jan'y-March, 1895, (reprint from Pacific Coast Dentist).
† International Dental Journal, February, 1895.
‡ Items of Interest, February, 1895.

### "THE NEW ERA."

Dr. W. G. A. Bonwill, at a meeting of the New York Odontological Society,* read a *resume* of his former paper bearing the above title, in order to bring it before the society for discussion.

The paper and the author were both criticised rather severely by Drs. Lord and Perry. Dr. Lord said that what was good in the paper would take care of itself, but that it seemed necessary to say something about that which was bad, or not so good. He thought that some of the statements in the paper were contradictory, and that too much importance was placed upon the use of plastics, the very high value put upon amalgam being counter to the views of a large majority of the profession.

Dr. Perry regretted that Dr. Bonwill does not discriminate more, instead of making such unqualified statements.

Dr. S. B. Mills thought that Dr. Bonwill was entirely right from his standpoint, Dr. Perry also being right from *his* standpoint; that there is a golden medium in practice, and that amalgam, as it can be used in the hands of men who have the requisite ability, is of great advantage to both operator and patient.

Dr. Jarvie thinks that a large share of the failures we meet with are due to the poor structure of the teeth treated, and to environment, rather than to incompetent operators. In frail teeth of young patients he approves of filling approximal cavities and the space between the teeth, with gutta-percha, the time that it is left in, depending upon circumstances. He thinks the paper will do good by calling attention strongly to certain points to which no attention would be paid if put in a mild way.

In closing the discussion, Dr. Bonwill defended his position with his usual energy and decision of statement. He regretted that gentlemen were so very careful of what they might say about the use of amalgam in their own practice that they would not rise to the discussion. He said: "You all speak as if you seldom use amalgam, and yet every one of you, I know, uses it. I know just how much amalgam is used by others, and I am willing to bring my practice to show you how much good I am doing with it."

* International Dental Journal, February, 1895.

### A COMBINATION BOX

Dr. Sailor exhibited before the New York Odontological Society* a combination of conveniencies calculated to add to the perfection of an operation, and to the ease and comfort of both patient and operator. It contains two compartments for hot water, at different temperatures—one for sterilizing purposes and one suitable for molding, modeling compound for impressions; also a glass of water held in place at one side by springs; two compartments for gutta-percha, requiring different grades of heat, and which can be kept ready for days without losing any of its qualities. There is a wire frame for holding the mouth mirrors at such a temperature that the breath will not condense upon them ; a depression for holding the instruments for using gutta-percha; a little slab for medicines for use in sensitive teeth, giving both the soothing effect of warmth and the effect of the medicines.

The entire stands on a bracket six or eight inches long, near the cabinet, always ready for use.

### THE CODE OF TEHICS.

At the June meeting of the Connecticut Valley Dental Society, the question of legitimate advertising by members of dental societies was discussed, and a committee appointed to report on the interpretation of the Code. The report was presented at an adjourned meeting, held in October.†

The committee submitted that whether dentistry is a profession or a trade depends on the course pursued by the individual practitioner, and the manner in which his business is conducted. There is nothing in the Code that calls for any sacrifice on the part of any practitioner who wishes to be known as a reputable professional man. The committee found no reason for suggesting any changes, either in the wording or in the construction of the Code, and no interpretation of its provisions necessary.

* International Dental Journal, Feb. '95

† Dental Cosmos, February, 1895.

EDITORIALLY.

Dr. Geo. J. Dennis* considers the probable causes of the apathy too often manifested in dental societies, and offers suggestions as to the methods of examination into the possible sources of this periodic trouble and remedies for the same.

The question of "Politics in Dental Associations"† and the baneful effect of political methods upon what should be purely professional and scientific work is considered. The American Dental Association is cited as an illustrious example.

The subject of "Legal Restriction of Nostrums" is considered‡ the ghastly record of fatalities consequent upon the ignorant use of secret cocain preparations demanding that the use of this drug be placed on a clearly-defined, legitimate basis. The margin of safety in the use of cocain, in accurately-known doses is so narrow that its use in unknown quantities, by men who are ignorant of the most elementary features of the action of the drug, in the nostrums vended for *painless dentistry*, falls little short of criminality, lacking only the feature of statutory definition to make it so. The proviso-clause in many State laws, permitting the "extraction of teeth" does not include the injection of unknown drugs in unknown quantities into the human system, and a strict interpretation of the clause would put an end to the *painless dentistry* business, with its grave menace to life and health.

* Dental Review, Jan. 15, 1895.
† International Dental Journal, Feb. 1895.
‡ Cosmos February. 1895.

